# Peripheral Red Blood Cell Split Chimerism as a Consequence of Intramedullary Selective Apoptosis of Recipient Red Blood Cells in a Case of Sickle Cell Disease

**DOI:** 10.4084/MJHID.2014.066

**Published:** 2014-11-01

**Authors:** Marco Marziali, Antonella Isgrò, Pietro Sodani, Javid Gaziev, Daniela Fraboni, Katia Paciaroni, Cristiano Gallucci, Cecilia Alfieri, Andrea Roveda, Gioia De Angelis, Luisa Cardarelli, Michela Ribersani, Marco Andreani, Guido Lucarelli

**Affiliations:** 1 International Center for Transplantation in Thalassemia and Sickle Cell Anemia, Mediterranean Institute of Hematology, Policlinic of the University of Rome “Tor Vergata” Italy; 2 Department of Biopathology and Diagnostic Images, Polyclinic of Tor Vergata Foundation, Rome, Italy; 3 Laboratory of Immunogenetics and Transplant Biology, Mediterranean Institute of Hematology, Policlinic of the University of Rome “Tor Vergata”

## Abstract

Allogeneic cellular gene therapy through hematopoietic stem cell transplantation is the only radical cure for congenital hemoglobinopathies like thalassemia and sickle cell anemia. Persistent mixed hematopoietic chimerism (PMC) has been described in thalassemia and sickle cell anemia. Here, we describe the clinical course of a 6-year-old girl who had received bone marrow transplant for sickle cell anemia. After the transplant, the patient showed 36% donor hematopoietic stem cells in the bone marrow, whereas in the peripheral blood there was evidence of 80% circulating donor red blood cells (RBC). The analysis of apoptosis at the Bone Marrow level suggests that Fas might contribute to the cell death of host erythroid precursors. The increase in NK cells and the regulatory T cell population observed in this patient suggests that these cells might contribute to the condition of mixed chimerism.

## Introduction

Allogeneic cellular gene therapy through hematopoietic stem cell transplantation is the only radical cure for congenital hemoglobinopathies like thalassemia and the sickle cell anemia.^1^ Persistent mixed hematopoietic chimerism (PMC) has been described in thalassemia.^2^,^3^ Recently, a split chimerism of the peripheral red blood cells was also described four years after transplantation.^3^,^4^ PMC provides a unique opportunity to perform a direct side by side comparison of normal and sickle erythropoiesis. However, the minimum proportion of donor cells that defines PMC differs in sickle cell disease (SCD) and thalassemia patients transplanted; and the cell populations, total leukocytes, mononuclear cells, or lineage-specific cells assayed for chimerism, also varies. The threshold percentage of donor cells sufficient to ameliorate the hemoglobin disorders has not yet been firmly established. In thalassemic patients after myeloablative HSCT, 10% to 20% of donor cells has been shown to be curative.^3^,^5^

Several potential factors seem to be associated with PMC. Less-intensive conditioning regimens are associated with a greater proportion of PMC. As just recently reported, T regulatory cells (Treg) and natural killer (NK) populations may help to establish persistent mixed chimerism.^6^,^7^ HLA-mismatched transplants in mice and humans demonstrate that donor NK cells target host hematopoietic tissue, eliminating host antigen-presenting cells, host hematopoiesis, and host leukemia. These effects translate into better engraftment, diminished risk from acute graft versus host disease (GVHD), reduced relapse from an NK-mediated graft-versus-leukemia effect and lower rejection rates.^8^–^10^

Recent studies suggest that type 1 regulatory cell clones of both donor and host origin can inhibit the function of effector T cells of either donor or host origin in vitro.^6^ These results suggest that Treg cells could be associated with PMC.

Normal homeostasis of the erythropoietic system requires an appropriate balance between the rate of erythroid cell production and red blood cell destruction. Growing evidence indicates that apoptotic mechanisms play a relevant role in the control of erythropoiesis under physiologic and pathologic conditions.^11^ We hypothesized that Fas might contribute to the cell death of SS erythroid precursors. The two questions, how two different erythroid populations may exist together during erythropoiesis in the bone marrow of PMC patients and if T, B, or other lymphocyte subsets, are responsible for allowing this persistent and stable chimerism, remain to be answered.

## Methods

### Transplant Protocol

According to the clinical protocol approved by the local institutional review board, the patient received BM from her HLA-matched healthy sister (Hb AA) after a conditioning regimen based on 14 mg/kg busulfan (Bu), 200 mg/kg cyclophosphamide (Cy), and 10 mg/kg anti-thymocyte globulin (ATG). For prophylaxis against GVHD, the patient received cyclosporine (starting on day −2) and short methotrexate (MTX) (10 mg/m^2^ on post-transplant days 1, 3, and 6 with folinic acid rescue). The course after allogeneic hematopoietic stem cell transplantation was uneventful, with the rapid hematologic engraftment and no signs of acute or chronic GVHD. The clinical characteristics of the patient and donor, and the regimen used in the preparation for the transplant are summarized in Table 1.

### Laboratory tests

#### Chimerism analysis of nucleated cells and burst-forming unit-erythroid colonies

Peripheral blood and bone marrow samples were collected in EDTA on days 20, 60, and 180 after the transplant, and thereafter during the annual routine follow-up examinations. DNA samples were extracted using the QIAamp DNA Blood Mini Kit (Qiagen, Valencia, CA, USA) or an automatic DNA extractor (Promega, Madison, WI, USA). The DNA was typed by short tandem repeats (STR) and the amelogenin locus using the AmpFISTR Profiler Plus kit (Applera, Foster City, CA, USA). Amplification reactions were carried out using 1–2 ng of input DNA following the manufacturer’s recommendations. Polymerase chain reaction products were run on an ABI Prism 3130xl Genetic Analyzer (Applera, Foster City, CA, USA). Informative loci in post-transplant samples were screened to quantify the percentage of donor cells in mixed chimeras. HSCT engraftment was quantified using fluorescent polymerase chain reaction primers for human identity markers based on the ratio between the peak areas of donor and recipient alleles. The mean value obtained after performing calculations for each informative STR was taken as the percentage of mixed chimerism. Burst-forming unit-erythroid (BFU-E) colonies were grown in agar and picked out singly for STR evaluation.^2^

#### Clonogenic assay

Assays for clonogenic hematopoietic progenitors were performed in methylcellulose semisolid cultures. Briefly, 1–2×10^5^ low-density bone marrow/peripheral blood cells were plated in duplicate in 35-mm tissue culture dishes, and suspended in 1 mL methylcellulose medium supplemented with stem cell factor, granulocyte/macrophage colony-stimulating factor, interleukin-3, and erythropoietin (Methocult GFH4434, Stem Cell Technologies, Vancouver, British Columbia, Canada). Cultures were incubated at 37°C in a fully humidified atmosphere containing 5% CO_2_. Plates were scored for BFU-E growth after 14 days of incubation. Using an inverted microscope, individual colonies were picked up from the Petri dishes and dispersed to single cell suspensions in 100 μL saline to assess the donor/recipient origin of the individual colonies using STR.

#### Chimerism of red blood cells

For cytofluorimetric analysis, red blood cells (RBCs) were washed and diluted in saline (0.5% final dilution). Five microliters of cell suspension were incubated with anti-ABO and anti-C, -c, -D, -E and -e monoclonal antibodies, following the manufacturer’s instructions (ABH- and RH- Erythrokit, Institute Jacque Boy SA, Reims, France). After the incubation, cells were washed with phosphate-buffered saline (PBS) and incubated with fluorescein isothiocyanate-conjugated anti-human immunoglobulin. After the incubation and two additional washes, the analysis was performed using a FC500 flow cytometer and transferred to the CXP analysis program (Beckman-Coulter Hialeh, FL, USA).

#### Evaluation of FAS and Treg in bone marrow and peripheral blood mononuclear cells

Whole blood and bone marrow specimens were obtained to evaluate T, B, NK, and FAS on erythroblasts: anti-CD3 FITC, anti-CD4 APC, anti-CD8 PE, anti-CD45 PercP Cy 5.5, and anti CD45 RA PE Cy7 were mixed in the first tube; anti-CD16 FITC, anti-CD56 PE, anti-CD45 PercP Cy5.5, anti CD3 PE Cy7, and anti CD19 APC in the second tube; and anti-CD95 PE, anti-CD71 FITC and anti-CD45 PercP Cy5.5 in the third tube. A volume of 10 μl of these MoAb cocktails (BD, Becton Dickinson, San Diego, C.A., USA) were combined with 100 μl of blood for 10 minutes at room temperature, then lysed with BD Pharm Lyse 1× for 20 minutes at room temperature and washed with 2% PBS plus bovine serum albumin (BSA). Samples were analysed with BD FACS Canto II and the software, BD FACSDiva. We evaluated co-expression of CD39 in CD4+CD25 high T cells for analysis of T reg cells. In humans, CD39 is a surface marker expressed almost exclusively by Foxp3^+^ cells.^12^ We also compared Treg data of the patient with Treg data of not transplanted SCD patient who was matched for age and sex.

#### Cytometric assay for erythroid cell precursors

We previously developed a flow cytometric assay to identify stage-specific erythroblasts directly in hematopoietic tissue (bone marrow) based on their expression of the transferrin receptor (CD71), which declines with erythroblast maturation. However, the decline in CD71 appeared to be gradual, without the formation of well-resolved subpopulations. In this study, we distinguished well resolved erythroblast subpopulations by considering, in addition to CD71, the forward scatter (FSC) parameter. FSC is a function of cell size and has been used previously to assess erythroblast maturation independently of cell surface marker expression. When the cells are analysed using both CD71 and FSC parameters, they consistently resolve into three principal subpopulations, which we labelled Ery A, Ery B, and Ery C erythroblasts. Ery A (CD71^high^ FSC^high^) are basophilic; Ery B (CD71^high^ FSC^low^) are late basophilic and polychromatic; and Ery C (CD71^low^ FSC^low^) are orthochromatic erythroblasts and reticulocytes.

We examined potential apoptotic regulators of erythroblasts. Fas (CD95) has been detected on cultured erythroblasts. We also compared Fas data of the patient with Fas data of not transplanted SCD patient who was matched for age and sex.

We examined whether Fas is coexpressed within the same cells by labelling bone marrow cells simultaneously with antibodies directed against Fas, as well as CD71. We also examined CD95 expression in the RBC of the recipient and donor at bone marrow and peripheral blood levels.

## Results

**Figure 1:**
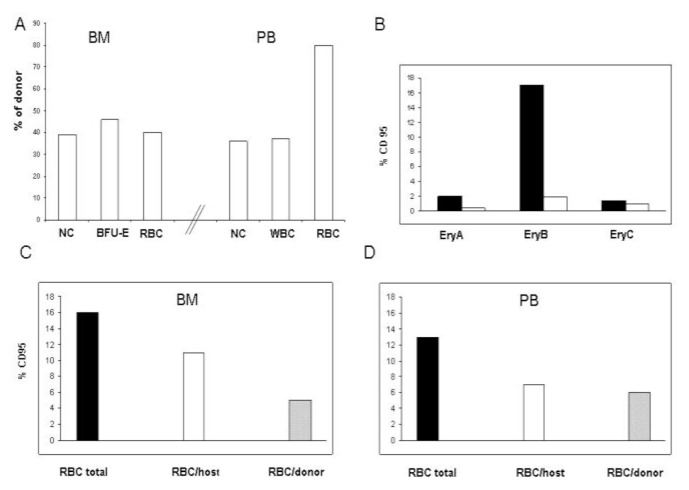
Comparison of bone marrow and peripheral blood donor chimerism and expression of Fas (CD95+) on erythroid precursors and red blood cells. **(A)** The percentages of donor engraftment in the bone marrow (BM) and peripheral blood (PB) four years after transplantation. NC= Nucleated Cells; RBC=Red Blood Cells; BFU-E= Burst-Forming Unit Erythroid. **(B)** The expression of Fas (CD95) on erythroid precursors in the patient ( black) vs. SCD control patient (white). **(C and D)** The expression of Fas (CD95+) on host RBC (white bar) and donor RBC (grey bar) in the BM (C) and PB (D).

Molecular analysis of sorted cell subgroups revealed mixed chimerism in nucleated cells, CD34+ progenitors, and RBCs in the PB and BM. Four years after transplantation, the level of donor nucleated cells (NC) was 39% in PB and 36% in the BM in parallel with a very high proportion of donor-derived RBCs (80%) in the PB. The proportion of donor-derived RBCs and BFU-E in the BM was 40% and 46%, respectively, indicating the presence of quantitatively different red cell/nucleated cell chimerism. (Figure 1).

Hb electrophoresis on peripheral blood four years post-transplantation showed HbA1 97,2 % Hb A2 2,8 %, without any traces of HbS.

The same haemoglobin phenotypes were observed on bone marrow examination. The value of HB post-transplant was 13 g/dl.

We also examined potential apoptotic regulators of erythroblasts, evaluating Fas (CD95) expression on cultured erythroblasts and red cells from BM and PB. We found that Fas was expressed by a significantly higher proportion of host erythroblasts, especially in late basophilic and polychromatic erythroblasts, and in host RBCs from BM (Figure 1).

**Figure 2:**
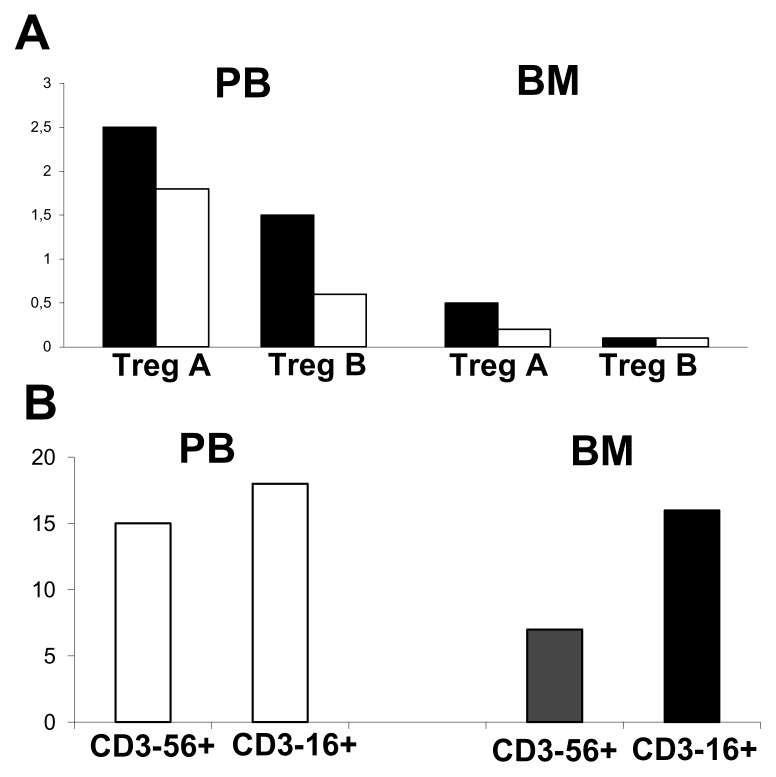
Treg cells and NK cells **(A)** Black bars indicate patient ; white bars indicate the control SCD. T reg A indicates CD4+CD25+CD127− T cells; T reg B, CD4+CD25+CD127-CD39+CD45Ra− T cells; PB. peripheral blood; and BM, bone marrow. **(B)** The percentage of NK cells in PB and BM in the patient.

An increase in Treg was also observed in the patient with respect to the control. From BM, we observed an increase in cytotoxic CD3-CD16^+^ NK cells (Figure 2).

## Discussion

In the hematopoietic system, the percentage of donor NCs correlates with that of erythroid cells, consistent with the current understanding of myelo- and erythropoiesis deriving from common myelo-erythroid progenitors. Furthermore, the percentage of donor RBC in the PB was much higher, supporting the hypothesis that donor and host erythroid precursors were at a competitive disadvantage for generating mature red blood cells. A possible explanation for the presence of a greater proportion of donor-derived RBC may be the improved survival of donor erythroid late precursors compared with the host counterparts, which might be destroyed during ineffective erythropoiesis. Apoptosis is an important mechanism by which ineffective erythroblasts are cleared within the intramedullary space, and our data suggest that Fas might contribute to the cell death of host erythroid precursors. The high level of polymerization of the sickle hemoglobin in host RBCs as well as in the host, early and basophilic, normoblasts might also determine mechanical defects that in turn increase the host cells’ susceptibility to clearance and loss.

The higher percentages of CD3-CD16^+^ NK cells in a mixed chimerism patient may play a role in control of host host-cell escape and in maintaining the chimerism condition. Pioneering studies by Velardi and colleagues revealed that patients with acute myelogenous leukemia transplanted from an NK alloreactive donor benefited from higher rates of engraftment and reduced rates of GVHD.^8^ The virtual abrogation of GVHD may be a consequence of NK cell-mediated killing of recipient antigen-presenting cells.^9^,^10^ The beneficial effects could also be related to depletion of patient antigen-presenting cells and facilitation of engraftment as a result of the killing of T cells, removing patient lymphohematopoietic cells, and production of growth factors required for engraftment and for accelerating recovery of myelopoiesis.

The increase in Treg populations, especially in peripheral blood, suggests that these cells may play an important role in sustaining long-term tolerance in vivo.

Our observation, confirming the presence of split chimerism between RBCs and their erythroid precursors, supports the concept that a limited HSC engraftment can provide a sufficient amount of normal hemoglobin and mature erythrocytes, and that this limited engraftment can inhibit the expansion of HbS-erythropoiesis.

Our results support the evidence that low levels of donor engraftment can result in significant functional improvement for patients with SCD. The observation that a few engrafted cells are sufficient to clinically control patients with SCD is particularly interesting in light of a possible gene therapy approach or stem cell transplantation in adult patients.

## Figures and Tables

**Table 1:** Clinical characteristics of the patient and transplantation

Patient Diagnosis	SCD
Age at transplantation/sex	Two years/F
HB at diagnosis	6 g/dl
N^∘^ of transfusions pre transplantation	2
Hb chain chromatography	α/β 0.94 α/γ+β 0.94 βs %100
Donor Sickle genotype/sex/age	Healthy/F/10 years
Donor HLA A B C DR/relationship	Identical/sister
Pt/Donor ABO type	0 Rh neg/A Rh pos
Pt/Donor subgroups	CcDeekk/ccdeekk
Transplant conditioning regimen	BU14 CY 200 ATG 10
Stem cell source	BM
GVHD prophylaxis	CsA/Methylpred/sMTX

SCD: sickle cell disease; Bu: busulfan; CY: cyclophosphamide; ATG: thymoglobulin; BM: bone marrow CsA: cyclosporine; Smtx: short methotrexate

## References

[1] Lucarelli G, Gaziev J, Isgrò A, Sodani P, Paciaroni K, Alfieri C, De Angelis G, Marziali M, Simone MD, Gallucci C, Roveda A, Saltarelli F, Torelli F, Andreani M (2012). Allogeneic cellular gene therapy in hemoglobinopathies--evaluation of hematopoietic SCT in sickle cell anemia. Bone Marrow Transplant.

[2] Andreani M, Testi M, Gaziev J, Condello R, Bontadini A, Tazzari PL, Ricci F, De Felice L, Agostini F, Fraboni D, Ferrari G, Battarra M, Troiano M, Sodani P, Lucarelli G (2011). Quantitatively different red cell/nucleated cell chimerism in patients with long-term, persistent hematopoietic mixed chimerism after bone marrow transplantation for thalassemia major or sickle cell disease. Haematologica.

[3] Hsieh MM, Wu CJ, Tisdale JF (2011). In mixed hematopoietic chimerism, the donor red cells win. Haematologica.

[4] Andreani M, Testi M, Battarra M, Lucarelli G (2011). Split chimerism between nucleated and red blood cells after bone marrow transplantation for haemoglobinopathies. Chimerism.

[5] Walters MC, Patience M, Leisenring W, Rogers ZR, Aquino VM, Buchanan GR, Roberts IA, Yeager AM, Hsu L, Adamkiewicz T, Kurtzberg J, Vichinsky E, Storer B, Storb R, Sullivan KM, Multicenter Investigation of Bone Marrow Transplantation for Sickle Cell Disease (2001). Stable mixed hematopoietic chimerism after bone marrow transplantation for sickle cell anemia. Biol Blood Marrow Transplant.

[6] Serafini G, Andreani M, Testi M, Battarra M, Bontadini A, Biral E, Fleischhauer K, Marktel S, Lucarelli G, Roncarolo MG, Bacchetta R (2009). Type 1 regulatory T cells are associated with persistent split erythroid/lymphoid chimerism after allogeneic hematopoietic stem cell transplantation for thalassemia. Haematologica.

[7] Isgrò A, Marziali M, Sodani P, Gaziev J, Erer B, Polchi P, Paciaroni K, Roveda A, De Angelis G, Gallucci C, Alfieri C, Simone MD, Zinno F, Isacchi G, Adorno G, Lanti A, Leti W, Aiuti F, Fraboni D, Andreani M, Lucarelli G (2010). Immunohematologic reconstitution in pediatric patients after T cell-depleted HLA-haploidentical stem cell transplantation for thalassemia. Biol Blood Marrow Transplant.

[8] Aversa F, Terenzi A, Tabilio A, Falzetti F, Carotti A, Ballanti S, Felicini R, Falcinelli F, Velardi A, Ruggeri L, Aloisi T, Saab JP, Santucci A, Perruccio K, Martelli MP, Mecucci C, Reisner Y, Martelli MF (2005). Full haplotype-mismatched hematopoietic stem-cell transplantation: a phase II study in patients with acute leukemia at high risk of relapse. J Clin Oncol.

[9] Shlomchik WD, Couzens MS, Tang CB, McNiff J, Robert ME, Liu J, Shlomchik MJ, Emerson SG (1999). Prevention of graft versus host disease by inactivation of host antigen-presenting cells. Science.

[10] Della Chiesa M, Vitale M, Carlomagno S, Ferlazzo G, Moretta L, Moretta A (2003). The natural killer cell mediated killing of autologous dendritic cells is confined to a cell subset expressing CD94/NKG2A, but lacking inhibitory killer IG-like receptors. Eur J Immunol.

[11] Wu CJ, Krishnamurti L, Kutok JL, Biernacki M, Rogers S, Zhang W, Antin JH, Ritz J (2005). Evidence of ineffective erythropoiesis in severe sickle cell disease. Blood.

[12] Borsellino G, Kleinewietfeld M, Di Mitri D, Sternjak A, Diamantini A, Giometto R, Höpner S, Centonze D, Bernardi G, Dell’Acqua ML, Rossini PM, Battistini L, Rötzschke O, Falk K (2007). Expression of ectonucleotidase CD39 by Foxp3+ Treg cells: hydrolysis of extracellular ATP and immune suppression. Blood.

